# Consensus on the terminologies and methodologies for masticatory assessment

**DOI:** 10.1111/joor.13161

**Published:** 2021-03-29

**Authors:** Thais Marques Simek Vega Gonçalves, Martin Schimmel, Andries van der Bilt, Jianshe Chen, Hilbert W. van der Glas, Kaoru Kohyama, Martine Hennequin, Marie‐Agnès Peyron, Alain Woda, Claudio Rodrigues Leles, Luciano José Pereira

**Affiliations:** ^1^ Department of Dentistry Federal University of Santa Catarina (UFSC) Florianópolis, Santa Catarina Brazil; ^2^ Department of Reconstructive Dentistry and Gerodontology / School of Dental Medicine University of Bern Bern Switzerland; ^3^ Division of Gerodontology and Removable Prosthodontics University Clinics of Dental Medicine University of Geneva Geneva Switzerland; ^4^ Department of Oral‐Maxillofacial Surgery, Prosthodontics and Special Dental Care University Medical Center Utrecht Utrecht The Netherlands; ^5^ Laboratory of Food Oral Processing Zhejiang Gongshang University Hangzhou China; ^6^ Food Research Institute National Agriculture and Food Research Organization (NARO) Tsukuba Japan; ^7^ University of Clermont Auvergne Clermont‐Ferrand France; ^8^ INRAE Centre, Human Nutrition Unit, Université of Clermont Auvergne Clermont‐Ferrand France; ^9^ Federal University of Goias (UFG) Goiânia, Goiás Brazil; ^10^ Federal University of Lavras (UFLA) Lavras Minas Gerais Brazil

**Keywords:** chewing, consensus, eating capability, food oral processing, mastication, terminology

## Abstract

A large number of methodological procedures and experimental conditions are reported to describe the masticatory process. However, similar terms are sometimes employed to describe different methodologies. Standardisation of terms is essential to allow comparisons among different studies. This article was aimed to provide a consensus concerning the terms, definitions and technical methods generally reported when evaluating masticatory function objectively and subjectively. The consensus is based on the results from discussions and consultations among world‐leading researchers in the related research areas. Advantages, limitations and relevance of each method are also discussed. The present consensus provides a revised framework of standardised terms to improve the consistent use of masticatory terminology and facilitate further investigations on masticatory function analysis. In addition, this article also outlines various methods used to evaluate the masticatory process and their advantages and disadvantages in order to help researchers to design their experiments.

## 1 BACKGROUND

For more than a century, the masticatory process has been thoroughly investigated, leading to a large number of reports in the literature, dating back to 1901.^1^ Several aspects of the masticatory function have been reported, such as masticatory physiology in dentate individuals,^2^, ^3^ food oral processing,^4^, ^5^, ^6^, ^7^ masticatory impairments after tooth loss^8^, ^9^ and improved masticatory function after different types of oral rehabilitation^10^, ^11^, ^12^, ^13^ or neurological disorders.^14^, ^15^ Several reviews on masticatory function have been published.^5^, ^6^, ^16^, ^17^, ^18^, ^19^, ^20^, ^21^ A good masticatory function is not only important for adequately fragmenting food in order to facilitate safe swallowing without choking, but it is also essential as masticatory impairments may have a negative effect on both digestion and nutrition.^22^, ^23^, ^24^, ^25^ Furthermore, mastication has a positive influence on brain function and cognition^26^, ^27^, ^28^, ^29^ and is an important factor in dental Patient Reported Outcomes (dPROs) like the dimensions ‘Oral Function’ or ‘Psychosocial Impact’ as well as Patient Satisfaction.^30^, ^31^

As listed above, literature shows a wide variation in methods and terminology. According to the Glossary of Prosthodontic Terms, ‘masticatory performance’ is defined as ‘a measure of the comminution of food attainable under standardised testing conditions’, while ‘masticatory efficiency’ is defined as ‘the effort required to achieve a standard degree of comminution of food’.^32^ That definition of masticatory performance is ambiguous since it comprises masticatory efficiency as well. In accordance with Bates et al (1976),^16^ that is (a) chewing performance, indeed refers to a state of chewing outcome following a particular number of chewing cycles, whereas (b) chewing efficiency denotes the number of chewing cycles needed to attain a particular chewing outcome. In other words, masticatory performance refers to the individual's ability to grind or pulverise a specimen of test food after a pre‐determined number of mastication cycles, while masticatory efficiency refers to the number of chewing cycles necessary to attain half the original particle size.^16^, ^33^, ^34^ However, there is a lack of consensus among researchers on the exact semantics of each term and similar terms are sometimes employed to describe different methodologies.^17^, ^35^ This may lead to comparisons among different test methods and therefore jeopardise scientific evidence on physiological or therapeutic protocols. For example, the term masticatory efficiency has been used interchangeably with the term masticatory performance,^36^ although they represent different tests of the masticatory function designed to produce distinct outcomes.

The aim of this consensus paper was to define the most commonly used terms and techniques related to evaluating masticatory function. Use of a common terminology will facilitate less ambiguous communication between researchers, clinicians and their patients. It will also enable better documentation and interpretation of research findings and clinical observations.

## 2 MATERIAL AND METHODS

The development of this document involved numerous experts in the masticatory function analysis. In the initial phase of the project, the team leaders (TMSVG and MS) selected a group of experts to discuss possible strategies to reach a consensus on terminology standards in masticatory function. A draft document containing the key terms was prepared and deliberated through email communications among authors. This first draft containing the definitions, advantages, limitations and clinical relevance was followed by an open discussion among participants. After a critical appraisal and individual feedback of each author, the reviews were collected and combined into one document, which was then shared among the authors. Once all the authors reached a final consensus, the revised manuscript was recirculated to the project participants for final comments and sign‐off for submission.

## 3 MASTICATORY FUNCTION ASSESSMENT

The outcome of mastication can be evaluated with two different approaches, the food bolus being collected either after a pre‐determined number of chewing strokes, or at the swallowing threshold, that is when the bolus is sufficiently cohesive and plastic to trigger swallowing. The research goals of these two approaches are different. When a subject is asked to chew and expectorate the food bolus after a fixed number of chewing strokes, the result reflects how well that subject performed in fragmenting or mixing the test food or other non‐nutritive test material (natural or synthetic). This parameter has been commonly referred to as masticatory performance or chewing performance.^16^, ^37^ In some studies, this procedure is called chewing test or C‐test.^20^ In the second approach, chewing until the subject is ready to swallow the food, other aspects of chewing are revealed.^38^, ^39^ In some studies, this approach is called mastication test or M‐test.^20^ The moment of swallowing depends on two major factors: the food textural and physical properties (ie hardness, stickiness, cohesiveness, moisture content, portion size) and oral as well as general physiological characteristics of an individual (ie dentition, biting/chewing force, tongue motility, salivary flow rate, age, neurological status, pain, intra‐oral sensitivity).^19^, ^38^, ^39^, ^40^, ^41^, ^42^, ^43^, ^44^, ^45^ It has been reported that persons having high masticatory performance (‘good chewers’) do not necessarily swallow their food after fewer strokes than persons having low masticatory performance (‘bad chewers’), although the more effective fragmentation capacity enables fewer chewing cycles needed to reduce food size for swallowing.^46^ Inversely, ‘bad chewers’ not necessarily swallow food long before ‘good chewers’ as they may swallow badly prepared food boluses. They may also refuse to swallow some types of food.^47^

**FIGURE 1:**
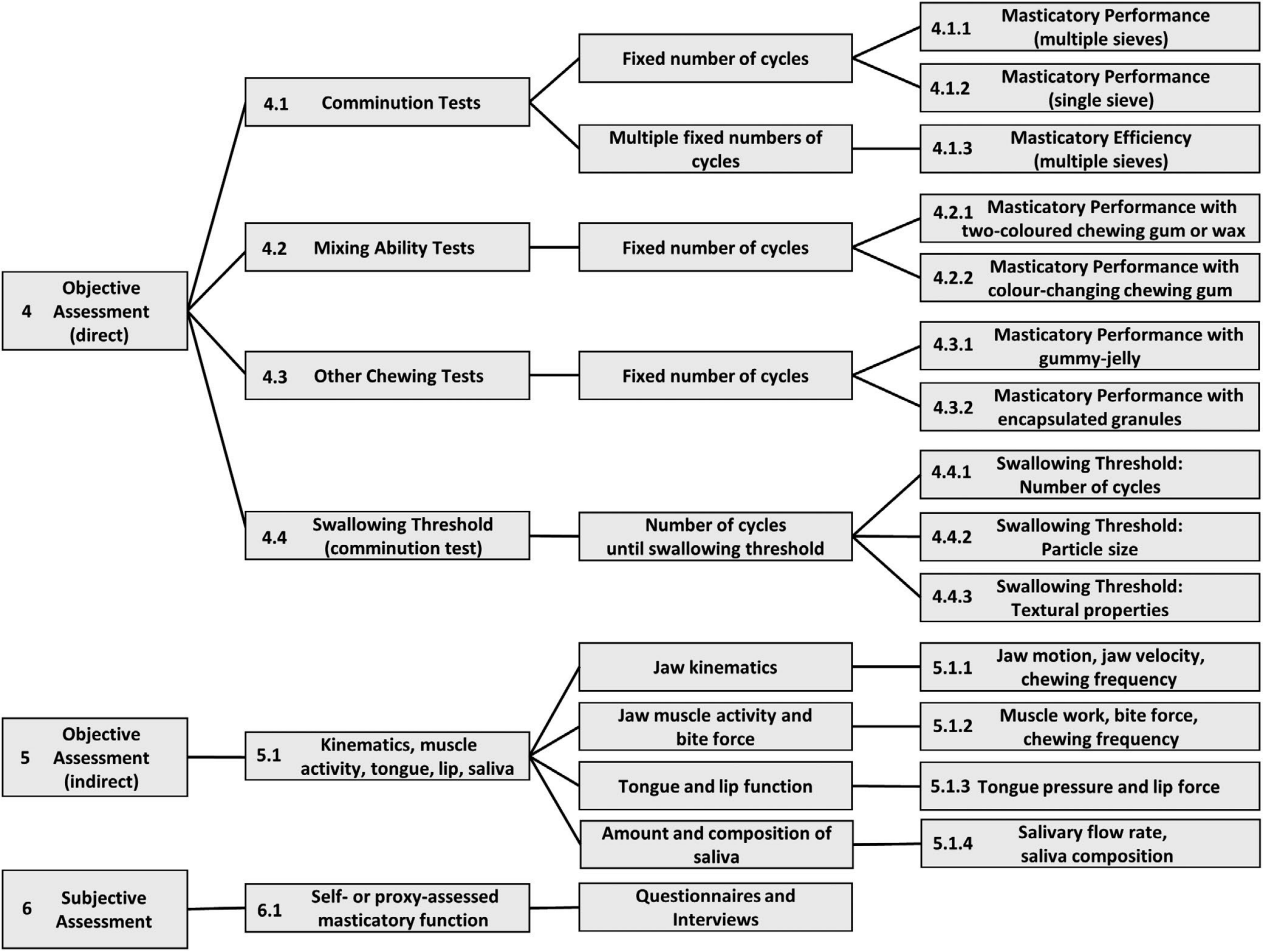
Summary of the masticatory function terms proposed

Masticatory function can be determined by many different methods. Each method yields different parameters which characterise the masticatory process. In this section, we give definitions, advantages, limitations and clinical relevance of the various methods and parameters (Figure 1):

## 4 OBJECTIVE ASSESSMENT (DIRECT ANALYSIS)

### 4.1 Comminution tests

A comminution test is performed on a brittle food such as nuts and raw carrots. Chewing will fragment the food, resulting in a collection of broken food particles constituting the food bolus. The food particles can be analysed by sieving or optical scanning. The chewed food bolus is then characterised by a distribution of particle sizes, usually expressed in the median particle size. If the food is chewed for a fixed number of chewing cycles, the result is defined as masticatory performance (or chewing performance).^16^, ^37^

#### 4.1.1 Masticatory performance with multiple sieves or optical scanning

The distribution of particle sizes reflects the masticatory performance (or chewing performance). Participants receive a portion of breakable artificial material (eg impression materials like Optosil/Optocal or hydrocolloid)^48^ or natural food (eg peanuts, almonds, carrots), which is chewed for a fixed number of chewing cycles, visually monitored by the examiner. The participant is then asked to expectorate the food bolus. After drying, the particles are sieved for 20 minutes through a stack of sieves with meshes generally ranging from 5.6 mm to 0.5 mm and a bottom plate.^49^ The distribution of bolus particle sizes by weight as obtained from the sieves can be described by a cumulative distribution function, the so‐called Rosin‐Rammler function.^50^ The function is characterised by the median particle size (X_50_ or D_50_) and the broadness of the distribution (b). The median particle size X_50_ is the aperture of a theoretical sieve through which 50% of the weight of the fragmented material could pass.^18^, ^37^, ^51^ The comminuted particles of the chewed food after a fixed number of chewing cycles can also be analysed by optical scanning.^49^, ^52^ The results obtained with optical scanning can be converted to a discrete particle size distribution, which again can be described by a cumulative distribution function.^52^

#### 4.1.2 Masticatory performance with single sieve

The degree of fragmentation of the chewed food or artificial test material is quantified by the percentage of the particles by weight that could pass through a sieve with a specific aperture after the food was chewed a fixed number of chewing cycles. This method is simpler and requires no further statistical analysis. However, the single sieve method is less reliable than the multiple sieve method especially if the sieve aperture is not close enough to the median particle size of the distribution of the chewed food.^53^ In summary, the use of more than one sieve will give more detailed information on the distribution of particle sizes of the chewed food as stated previously.^19^

#### 4.1.3 Masticatory efficiency with multiple sieves

Masticatory efficiency (or chewing efficiency) is defined as the number of chewing cycles needed to achieve a particular chewing outcome characterised by a median particle size (X_50_ or D_50_) that equals half of the particle size before chewing.^16^, ^33^, ^34^ Chewing efficiency can be calculated from a power function that describes the decrease of the median particle size as a function of the number of chewing cycles.^33^, ^54^ To perform such a calculation, the median particle size should be determined in multiple experiments, each experiment having a different number of chewing cycles. Thus, the masticatory efficiency test is a more elaborate version of the masticatory performance test with multiple sieves: it is based on several determinations of masticatory performance. Each determination after a different number of fixed chewing cycles.

##### Comminution tests: Advantages

**FIGURE 2:**
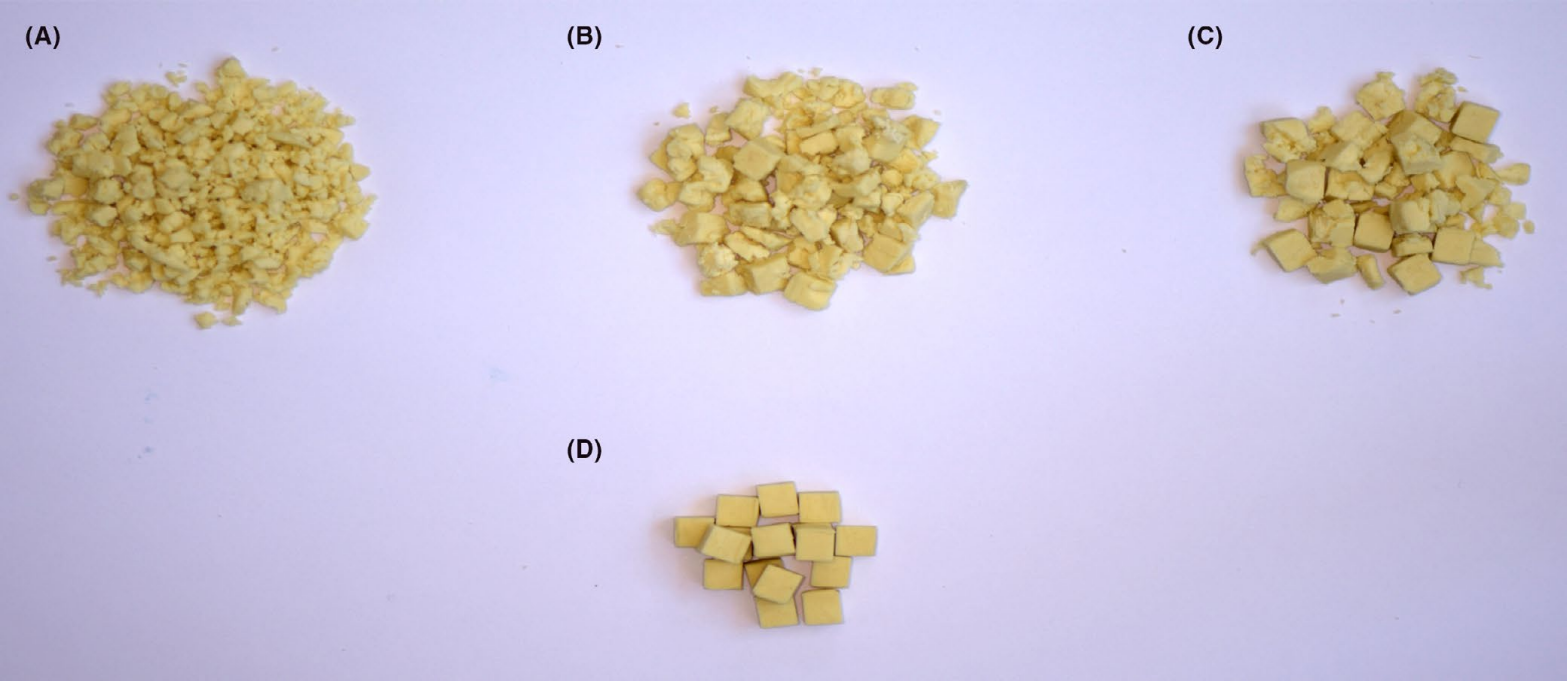
Masticatory performance analysed by a comminution test, using Optocal as test material and 20 masticatory cycles. A. Example of a good chewer; B. Example of a medium chewer; C. Example of a bad chewer; D. Cubes before chewing

The human masticatory apparatus is evolutionary developed to enable chewing brittle solid foods like nuts and carrots and tough foods like meat. An ability to chew a brittle solid test food will be concomitant with an ability to chew a broad spectrum of similarly hard or weaker types of foods. Shortly spoken, if one is able to chew fairly hard and tough foods, one is able to chew everything. If such a test is not feasible anymore for particular patient groups, then other tests are available with easier masticatory tasks, also allowing a differentiation of masticatory function in such patients. Furthermore, a solid test food includes all aspects of food comminution during chewing, that is transport, capturing and breakage of particles during each cycle, while mixing the food bolus with saliva. Comminution tests have been successfully used in numerous studies to quantify masticatory function. These tests enable the determination of both chewing performance as well as chewing efficiency. Furthermore, comminution tests can be used to determine parameters of chewed food just before swallowing as well. Comminution tests are a reliable way to quantify how well a person or a group is able to chew (Figure 2). For the masticatory efficiency, more detailed information of the chewing process is obtained as several tests after different numbers of chewing cycles are performed, offering a comparison of inter‐subject at the same stage of food comminution and constant intra‐subject and inter‐subject ratios between and within samples, respectively.^54^ Comminution tests are sensitive to changes in the oro‐facial system, since it is significantly related to maximum voluntary bite force as well as dental state.^9^

##### Comminution tests: Limitations

The limitations inherent to comminution tests refer to the selection of the appropriate test food/material for each type of population investigated (which also occurs with other tests described below). Care must be taken since the use of more rigid test materials such as Optosil may not be suitable for children and/or edentulous patients or patients with neuromuscular disorders. These individuals will not be able to adequately fragment the test food because their maximum bite force is lower than the force needed to break the test food particles and, therefore, differences within individuals will not be detected. The same occurs for very soft test foods for patients with excellent fragmentation capacity, since all the individuals evaluated will be able to fragment the food very well and subtle differences may go unnoticed. Furthermore, chewing brittle food may constitute an aspiration risk in dysphagic individuals such as stroke victims, very old institutionalised seniors or amyotrophic lateral sclerosis patients.

##### Comminution tests: Relevance

The comminution methods provide reliable parameters to quantify, for instance, the influence of dental treatment on chewing by measuring the performance before and after treatment in a group of patients.^55^, ^56^, ^57^, ^58^ These methods also facilitate the comparisons within and between subjects or groups of subjects or before/after rehabilitation, for example (Figure 2).

### 4.2 Mixing ability tests

**FIGURE 3:**
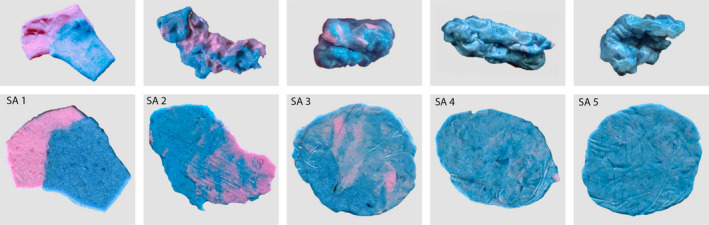
The Subjective Assessment (SA) scale for categorial evaluation of masticatory performance with a two‐coloured chewing gum (Hue‐Check Gum©).^60^, ^61^ SA 1: chewing gum not mixed, impressions of cusps or folded once, SA 2: large parts of chewing gum unmixed, SA 3: bolus slightly mixed, but bits of unmixed original colour, SA 4: bolus well mixed, but colour not uniform, SA 5: bolus perfectly mixed with uniform colour. Categories SA1 and SA2 would signify a severely reduced chewing function

**FIGURE 4:**
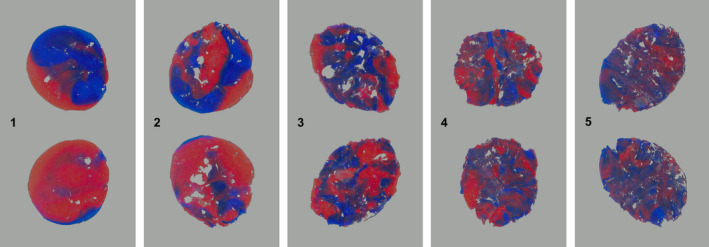
Masticatory performance determined from the mixing of the colours of the two‐coloured wax. Examples of digitised images of both sides of chewed and flattened two‐colour wax. 1: very badly mixed, 2: badly mixed, 3: intermediately mixed, 4: well mixed, 5: very well mixed

A non‐nutritive plastic test material is masticated for a given number of chewing strokes, and then retrieved from the oral cavity for analysis. The form and colour of the bolus might be evaluated. Subsequently, excess saliva is removed from the bolus, which is then placed in a transparent plastic bag and flattened to a wafer of typically 1mm thickness.^14^, ^38^, ^59^ Then, a digital image from both sides of the wafer is obtained under standardised lightning conditions.^60^ Therefore, the scanning of the wafer on a flatbed scanner lends itself to obtain standardised image capturing conditions (Figures 3 and 4). For the evaluation of dysphagic persons for whom the comminution tests might constitute a health hazard or for individuals with assumed severely impaired chewing function, alternative tests with cohesive test foods like chewing gum or wax have been developed.^14^, ^59^

#### 4.2.1 Masticatory performance with two‐coloured chewing gum or wax

For this test, pieces of two‐coloured wax or chewing gum (typically of blueish and reddish colour) are masticated for a given number of chewing strokes (most commonly n = 20).^59^, ^60^, ^61^, ^62^, ^63^ It was demonstrated that at 20 chewing strokes the tests show the best discriminatory characteristics between subjects or different oral conditions (Figures 3 and 4).^63^ The bi‐coloured specimens characterise the effect of the oro‐facial system on the bolus kneading with a quasi‐logarithmic decay pattern of the colours, depending on the characteristics of the specimens and the efficiency of the oro‐facial system to knead and form the gum bolus. A 5‐graded scale for a nominal assessment of colour mixture and bolus form has been proposed.^64^ Similar scales are still used, but often in combination with an opto‐electronic assessment of two‐coloured chewing.^61^, ^63^ There are many methods described that may be employed to assess the colour mixture among them the most commonly applied: Variance of Hue (VOH),^63^ intensity distribution of light,^65^or spatial heterogeneity.^66^, ^67^ The assessment of chewing performance with a mixing ability test correlates significantly with comminution tests at 20 chewing cycles.^59^, ^68^

#### 4.2.2 Masticatory performance with colour‐changing chewing gum

This method was mainly described by Japanese Researchers for the evaluation of masticatory performance (Evaluating Gum XYLITOL; Lotte Co., Ltd). The gum is chewed between 20 to 200 times and changes its colour from yellowish‐green to red, depending on the individual masticatory performance.^69^ The masticatory performance is measured by the colorimeter method using the L*a*b colour space model (CR‐13; Konica Minolta Sensing, Tokyo, Japan).^70^ However, a visual analysis based on a validated and reliable colour scale is also available.^69^

##### Mixing ability tests: Advantages

The mixing ability tests are very quick and cheap to perform. Hence, they demand very little time and effort in the scope of large test batteries or in cases of tested individuals with little resilience like patients with dementia.^15^, ^71^ Dysphagic patients like stroke or Amyotrophic Lateral Sclerosis patients may undergo this procedure without the risk of aspiration of particles.^14^ It has been recommended as the preferred method to assess masticatory function in subjects with pronouncedly impaired masticatory performance like complete denture wearers.^59^, ^72^ The nominal assessment scales allow for chair‐side and evaluation of the masticatory function, even for evaluators with little experience like wards in geriatric hospitals,^15^ and is suitable for research with children due to the pleasant taste. The various opto‐electronic assessment tools are often applied in research; however, the methods mostly need only a flatbed scanner and a computer for evaluation. First attempts for evaluation of the colour mixture with a smartphone application have been successful and will surely allow for further technical simplification in the near future.^63^, ^73^

##### Mixing ability tests: Limitations

Like any other test food, each type of gum or wax needs to be validated to understand the specific colour‐mixing characteristics. They may depend on the colours, the rheological characteristics, and hardness.^60^, ^63^ Furthermore, the multitude of opto‐electronic assessment tools make a comparison of results difficult between studies. Also, the material characteristics of chewing gum are very complex, as it changes hardness during the masticatory process, and this might further complicate standardisation of test food. There is little information if the colour of the chewed specimens’ changes after being exposed to saliva; hence, it is recommended to capture a digital image of the bolus or flattened wafer immediately after the clinical test. With the continuous improvement of integrated smartphone cameras this challenge might soon be resolved. Another limitation of this test is that chewing gums become soft and therefore easy to chew. In this sense, for individuals with good chewing capacity, the task of mixing colours is easily accomplished. Saturation of the mixture of colours occurs, making refinement between individuals with subtle differences in masticatory capacity inaccurate.^19^

As the mixing ability tests then depend less on the maximum available bite force (like in the case of granulometry) and rather evaluate the ability to form and knead a bolus, they might be less suitable for research questions that indirectly assess the increase or decrease of bite force.^74^

##### Mixing ability tests: Clinical relevance

There are numerous applications in which the mixing ability tests are successfully used. Firstly, in the quick and simple assessment of masticatory deficiencies in dental offices, hospitals or geriatric wards as part of a functional assessment of a person. Secondly, assessing masticatory performance with mixing ability tests may be used in large epidemiological studies with extensive test batteries as they only require very little time to perform on the patient and may be evaluated later after image capture. Thirdly, the use in the scope of clinical studies in settings where the granulometry tests cannot be conducted due to a lack of facilities and/or know‐how.

### 4.3 Other chewing tests

#### 4.3.1 Masticatory performance with gummy jelly

**FIGURE 5:**
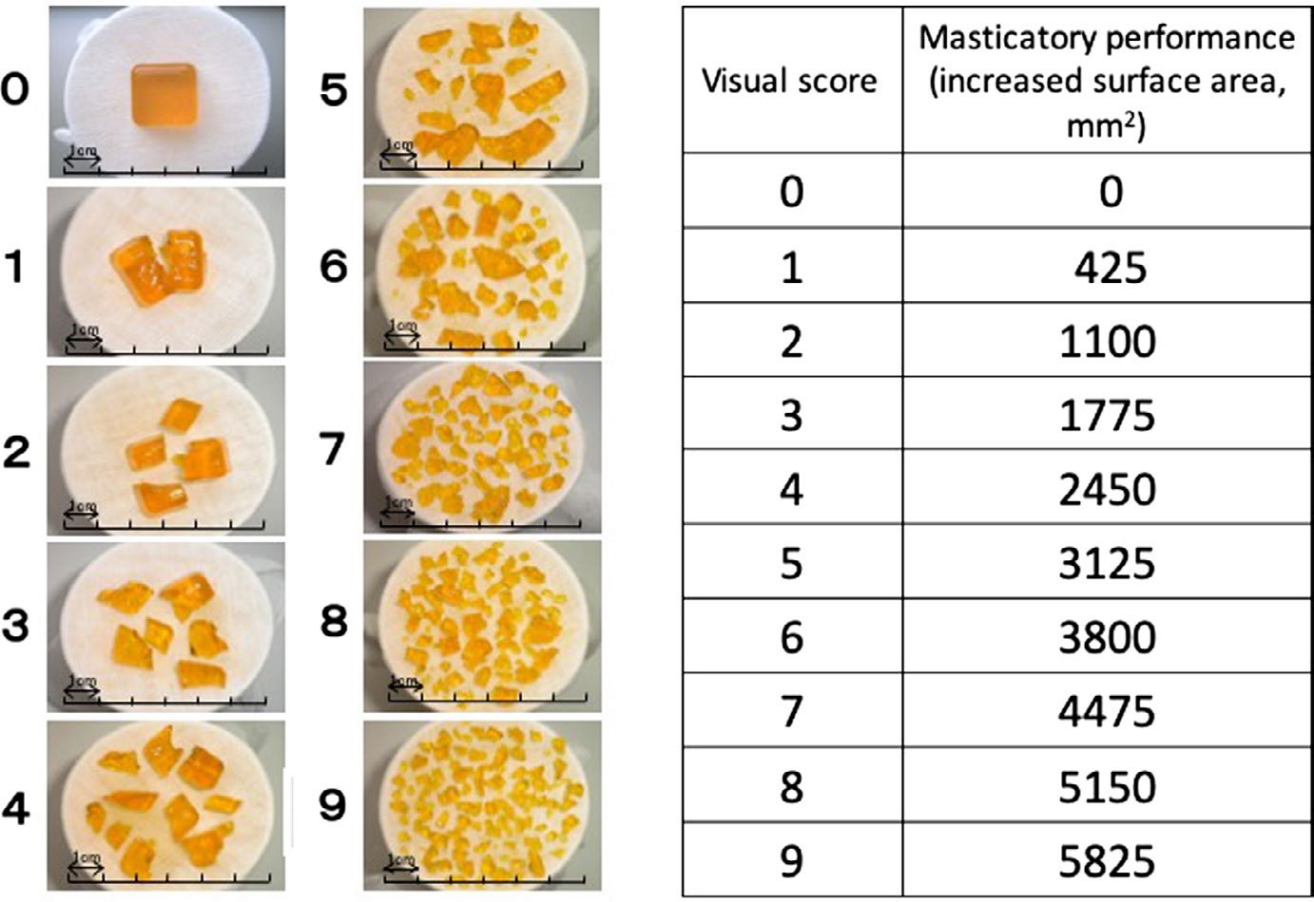
Masticatory performance measured using gummy jellies. Visual score from 0 (not chewed) to 9 (high performance) determined using 50 mg/dL gradations of glucose concentrations from a piece of gummy jelly.^80^

Gummy jelly is chewed for a pre‐determined number of cycles. The concentration of glucose or ß‐carotene dissolved in saliva is determined.^75^, ^76^, ^77^, ^78^ In addition, a new method was proposed based on the analysis of photographic image of the comminuted gummy jelly particles and the posterior calculous of the surface area (Figure 5).^79^, ^80^

#### 4.3.2 Masticatory performance with encapsulate granules

**FIGURE 6:**
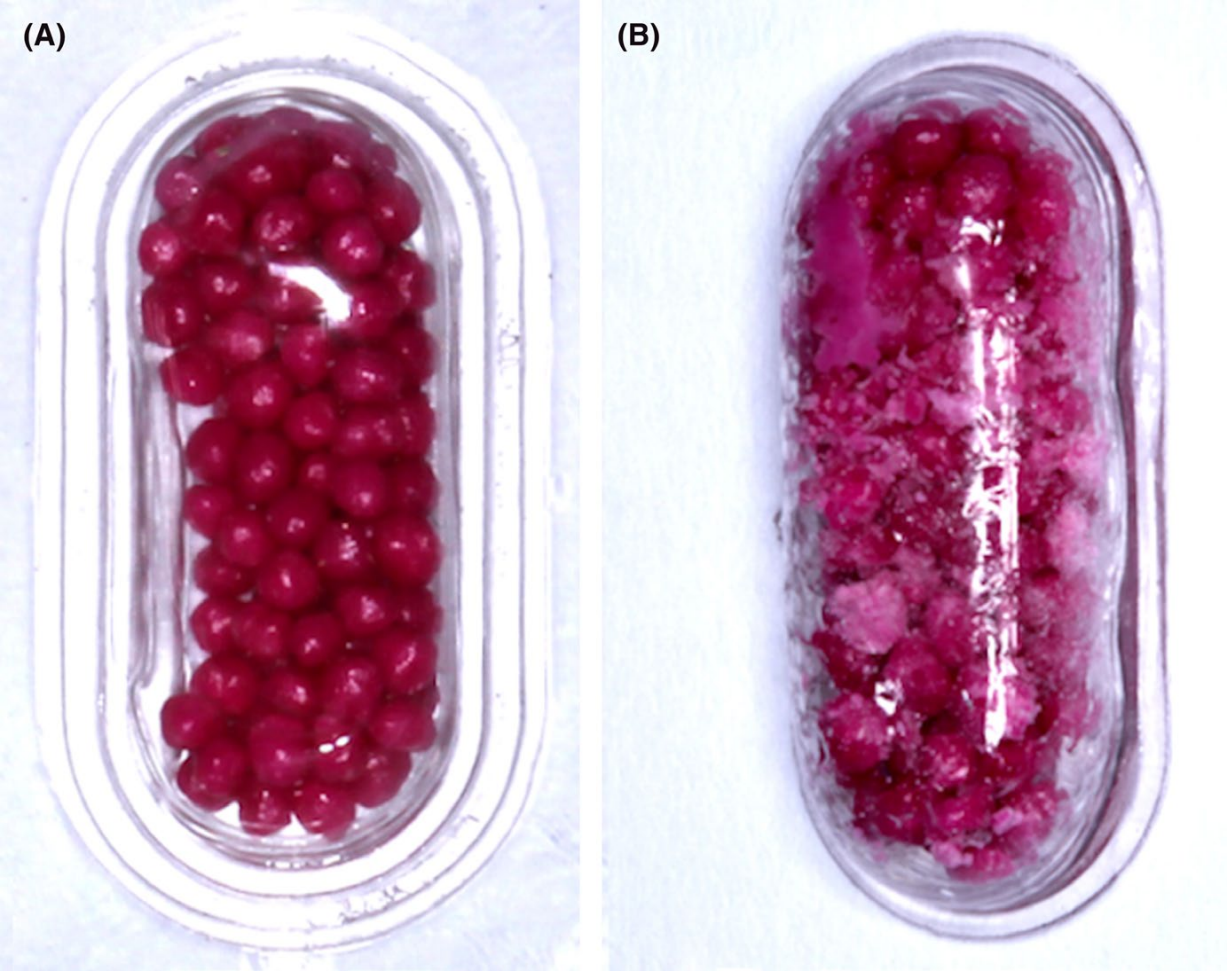
Masticatory performance analysed with encapsulate granules. A. Capsule appearance before chewing; B. Capsule appearance after chewing

These methods are based on the difference in dye concentration of fuchsine,^81^, ^82^ erythrosine^83^, ^84^ or adenosine triphosphate (ATP) among subjects after a mastication test. This method requires a spectrophotometer for this quantification. Pigment‐coated granules are prepared and sealed in a rubber or polymerising vinyl chloride (PVC) capsule (Figure 6). For fuchsine method, about 250 mg of fuchsine beads is used, while for erythrosine methods 730 mg of granules is required.^83^, ^84^ ATP methodology applies 5 g of granules. The individual is instructed to freely masticate a single capsule containing the pigment‐coated granules for a given number of cycles (Figure 6). Afterwards, the capsule is opened and its content is dissolved in water with constant stirring.^81^ The produced solution is filtered and the dye concentration is determined using a spectrophotometer.^84^ Each pigment must be analysed in a specific wave length (fuchsine at 546 nm,^81^ erythrosine at 565nm^83^, ^84^ ATP at 259 nm).

##### Other chewing tests: Advantages

These methods are easy to apply and measure a considerable number of subjects within a reasonable time period, with good reproducibility.^82^, ^83^ In regard to the spectrophotometer methods, the granules have stable physical properties and remain moisture‐proof within the rubber capsule.^83^ From the gummy jelly methods, it also represents a simple and non‐expensive method that obtains objective numerical values in a short time, using a relatively stable test material.^75^, ^83^ A portable equipment of analysis (Glucosensor GS‐II and the sensor tip, GC Japan) and a standard gummy jelly (glucose‐containing gummy jelly by GC Japan; beta‐carotene jelly by UHA Mikakuto Co., Ltd.) were also commercially available. The comminuted gummy jelly employing images evaluation has a close correlation with the fully automated method and can be a useful technique because of its accessibility under different conditions.^79^

##### Other chewing tests: Limitations

The rubber capsule is not representative of various foods sizes, shapes and stiffnesses; hence, only one aspect of mastication can be evaluated^83^ (since chewing an artificial single piece, which itself does not become crushed into particles, assumable does not feel natural, which reduces the validity and reliability of these methods in comparison to the comminution method, for instance).^82^, ^84^ Moreover, the use of capsules may include a type II error (failing to detect an effect that is present),^82^ which may explain the results of previous studies that found no differences after the conversion of a conventional complete denture into implant‐retained overdentures,^85^ or to detect any significant increase in the chewing performance after renewal of complete dentures.^86^ In regard to the gummy jelly methods, the collection and rinsing of comminuted gummy jelly, as well as the dissolution of the ingredients manually, needs proficiency of operating personnel and may thus affect measurement accuracy. However, a previous study had already established the appropriate set‐up conditions of the rinsing and dissolving operations for measuring masticatory function using a fully automatic measuring device.^75^

##### Other chewing tests: Clinical relevance

These methods are easy to apply in epidemiological studies, especially the rubber capsules, which are easy to chew by individuals with different dental conditions. Correlation between the mixing ability determined by colour changeable chewing gum and shearing ability determined by the gummy jelly was recently reported for community‐dwelling Japanese older adults.^87^ However, due to the low specificity of the capsules, additional masticatory analysis might be required. In the gummy jelly analysis, it is possible to use the smartphone camera for evaluating the surface area of the gummy jelly, making this technique more versatile and accessible.^79^

### 4.4 Swallowing threshold

The swallowing threshold is the moment that a person is ready to swallow the food. The condition of the food bolus at swallowing threshold is the cumulative result of bolus formation in all previous cycles.^88^, ^89^ Important swallowing threshold parameters can be obtained from comminution tests with natural foods (eg peanuts, almonds, carrots) or artificial (eg Optosil, Optocal) materials:

#### 4.4.1 Swallowing threshold number of cycles

The number of chewing cycles until the moment of swallowing.

#### 4.4.2 Swallowing threshold particle size

The median particle size of the food bolus just before swallowing.

#### 4.4.3 Textural properties of the chewed food bolus

Hardness, stickiness, cohesiveness, fibrous orientation, moisture content, particle adhesion, sufficient deformability of the bolus, sufficient internal consistency of particle content.

##### Swallowing threshold: Advantages

Swallowing is initiated when it is sensed that a batch of food particles is bound together under viscous forces so that it forms a bolus.^18^, ^38^ The moment of swallowing is triggered by characteristics of the food bolus, such as particle size, content of saliva incorporation, bolus viscosity, bolus cohesiveness and bolus flowability.^4^, ^18^, ^40^, ^41^, ^43^ Comminution tests at the swallowing threshold measure other aspects of chewing than masticatory performance tests (fixed number of chewing cycles).^43^ The number of chewing cycles needed to prepare food for swallowing was rather constant within a subject for one type of food,^89^ whereas large variations in the number of chewing cycles until swallowing were observed among subjects for one type of food.^46^, ^89^ Although the number of chewing cycles needed to prepare food for swallowing largely varied among healthy dentate people, this number was shown to be only weakly correlated with the chewing performance.^90^ Thus, a subject with a high masticatory performance does not necessarily swallow food after a smaller number of chewing strokes than a subject with a less high masticatory performance. It was reported that the upper limit of the median particle size of carrot particles swallowed by a group of young persons with good oral health was 4.0 mm.^47^ Declining masticatory function because of compromised dentition is responsible for swallowing poorly chewed food^46^, ^47^ and for chewing longer before swallowing.^90^, ^91^ Analysis of the food bolus at the swallowing threshold provides information of the textural properties of the food bolus.^40^ This knowledge can be useful in understanding the relationship between mastication and sensory perception ^5^, ^92^ or between mastication, digestion and nutrition.^93^, ^94^

##### Swallowing threshold: Limitations

Food characteristics have a large influence on both the number of chewing cycles and the food bolus characteristics at the swallowing threshold. Dry and hard food products require more chewing cycles before swallowing than moist and soft products.^5^, ^95^, ^96^ More time is needed to break the food down and to add enough saliva to form a cohesive food bolus suitable for swallowing. Also, the volume of the food largely influences oral physiology. For larger portion sizes, subjects needed more time and chewing strokes before they swallowed the food.^46^ Therefore, comparison of the parameters at the swallowing threshold of different groups of subjects can only be performed when using exactly the same consistency and volume of the test food.

##### Swallowing threshold: clinical relevance

Swallowing threshold tests determine the particle size distribution and textural properties of the chewed food bolus and the number of cycles needed to swallow. In this way, information is obtained on how persons normally swallow their food. This is especially important for patients with functional dyspepsia or the elderly, with compromised dentition since deficient comminution could produce a reduction of the gastric emptying rate, antral or fundal hypomobility, lack of antro‐pyloric‐duodenal co‐ordination and inhibition of intestinal feedback.^97^

## 5 OBJECTIVE ASSESSMENT (INDIRECT ANALYSIS)

### 5.1 Jaw kinematics, jaw muscle activity, bite force, tongue and lip function and saliva

Jaw movement and the neuromuscular control of the jaw muscles are important aspects of the food comminution. The basic rhythmic activity of the jaw‐opening and jaw‐closing muscles is known to be generated by a central pattern generator located in the brainstem.^98^ Adjustments of motor output in response to changes in food resistance are mediated by feedback from sensory receptors, such as mechanoreceptors in the periodontal ligament and muscle spindles in the jaw‐closing muscles.^99^, ^100^ Factors influencing the masticatory process are dentition, jaw muscle activity, bite force, tongue function and lip force.^19^ Furthermore, the production of sufficient saliva is indispensable for good chewing. The water in saliva moistens the food particles, whereas the salivary mucins bind masticated food into a coherent and slippery bolus that can be easily swallowed.^101^

#### 5.1.1 Jaw kinematics

Jaw movement can be recorded during chewing with a magnetic, an electromagnetic or an optical motion analysis system (eg Mandibular Kinesiograph (K7/SMS and JT3D, Articulograph, Optotrak)).^4^, ^102^, ^103^ Jaw velocity and jaw acceleration as well as the chewing frequency (the number of chewing cycles per minute) can be determined from the jaw gape signal. Jaw kinematics can also be analysed from video recording a chewing sequence.^104^

#### 5.1.2 Jaw muscle activity and bite force

Jaw muscle activity is commonly recorded from the masseter and anterior temporal muscles using bipolar surface electrodes.^4^, ^102^ Muscle activity can be obtained under dynamic conditions (chewing) or under static conditions (maximum voluntary clenching). From chewing experiments, muscle activity and muscle work can be determined for each chewing cycle.^4^, ^103^ Furthermore, the chewing frequency can be determined from the muscle activity bursts occurring during jaw closing. Maximum voluntary bite force is measured while participants clench as hard as possible on a bite force transducer placed between the molar teeth.^105^

#### 5.1.3 Tongue and lip function

Maximum tongue pressure is measured with a tongue pressure measuring device while subjects raised the tongue with maximal voluntary effort.^106^ Maximum tongue force can be obtained with a strain gauge mounted on a mouthpiece.^107^ Lip function can be determined from the so‐called lip‐length and snout indices^108^ or from maximum lip force measurements.^106^, ^109^

#### 5.1.4 Amount and composition of saliva

Mechanically stimulated salivary flow rate can be determined in a standardized way from chewing on a piece of tasteless Parafilm. Over a period of 5 min, a subject expectorates saliva at 30‐s intervals into a pre‐weighed container, and flow rate (mL/min) is then calculated. Flow rates of mechanically stimulated saliva between 0.52 and 4.55 mL/min have been reported for healthy subjects.^110^ Lately, an electronic tool to evaluate oral moisture (Mucus, Life Co., Ltd.) was introduced and is applied in the context of the diagnosis of oral hypofunction.^111^ The threshold for oral dryness was defined as a value of less than 27 as obtained with an oral moisture checker.^112^

##### Jaw kinematics, jaw muscle activity, bite force, tongue and lip function, and saliva: Advantages

Food properties such as structure, composition, appearance, volume, size and shape influence the masticatory process.^4^, ^102^, ^103^, ^113^, ^114^ Food properties had a small effect on the average chewing frequency during a chewing sequence.^103^ However, large significant differences in this parameter were observed between foods of different hardness at the beginning of a chewing sequence. In that phase, firm foods were chewed much slower than soft foods. A study on model foods showed that products with mainly plastic behaviour were chewed slower than elastic products.^113^ Muscular work was significantly influenced by both chewing phase and food type.^4^, ^103^ Studies on jaw kinematics and jaw muscle activity provide detailed information on the neuromuscular control of chewing.^100^, ^115^ The important role of saliva for chewing and swallowing was demonstrated by the finding that the number of chewing strokes, hence time in the mouth, needed for swallowing significantly increased after experimentally induced oral dryness.^64^ Amount and composition of saliva play an important role in oral processing and perception of the food.^92^

##### Jaw kinematics, jaw muscle activity, bite force, tongue and lip function, and saliva: Limitations

Studies on jaw kinematics and jaw muscle activity can only be performed in the laboratory. There is no ‘gold standard value’ of jaw kinematics. Chewing frequency depends on the mastication centre in the brain stem, which is specific to each subject. Therefore, chewing frequency cannot be used for inter‐subject comparisons.

##### Jaw kinematics, jaw muscle activity, bite force, tongue and lip function, and saliva: Clinical relevance

It was demonstrated that a lack of change in mean chewing frequency values could be used as a criterion for good masticatory neuromotor control, and alternatively, large variation in mean chewing frequency values could be indicative of an impaired masticatory function.^104^, ^116^

## 6 SUBJECTIVE ASSESSMENT

### 6.1 Self‐ or proxy‐assessed masticatory function (chewing ability)

Perceived or self‐assessed masticatory function, evaluating the quality of masticatory function,^117^, ^118^, ^119^ screening of masticatory disorders^120^ or analysing the food intake ability.^121^ Most instruments enable the patient to judge comfort discomfort when chewing.^17^, ^122^

Self‐ or proxy‐assessed masticatory function has been called masticatory ability in numerous studies. Masticatory ability is assessed from interviewing persons and filling out questionnaires on oral function. It can be quantified based on dichotomic answers (yes/no), 5‐point Likert scale (ranging from a score of 0 (very easy to chew) to a score of 5 (very difficult and avoided), categorical answers (‘never’, ‘sometimes’, ‘often’, ‘always’ or ‘somewhat difficult’, ‘moderately difficult’, very difficult’, ‘extremely difficult’) or Visual Analogue Scale (VAS) (with extremities varying from ‘not at all difficult’ to ‘impossible to chew’). In the literature, different validated instruments are available, such as:

#### 6.1.1 Quality of masticatory function (QMFQ)

This self‐applied questionnaire consists of 26 questions specifically related to the frequency and/or difficult on chewing different types of food during the two weeks before the evaluation.^117^, ^118^

#### 6.1.2 Chewing function questionnaire (CFQ)

The CFQ is a one‐dimensional instrument to measure the impacts of masticatory function in prosthodontic patients. The CFQ contains 10 items, which evaluate whether you have difficulty chewing different types of food, difficulty in biting different foods, if you feel insecure during chewing, if food sticks or catches on the teeth or dentures.^119^

#### 6.1.3 Screening for masticatory disorders in older adults (SMDOA)

The SMDOA is a 9‐question questionnaire designed to detect masticatory changes in community older adults. This epidemiological screening is capable of determining the initial diagnosis of masticatory disorder to be referred for diagnostic confirmation.^120^

#### 6.1.4 Food intake ability (FIA)

Subjects rate their chewing difficulty with foods of various textures and hardnesses. The questionnaire consists of 35 food items that are classified into five grades (seven items for each grade) based on the hardness, evaluating possible masticatory difficulties presented by the patients.^123^

#### 6.1.5 International classification of functioning, disability and health (ICF) for oral functions

This instrument is the most comprehensive model for describing human functioning in relation to health and the environment. It was adopted by the World Health Organization in 2001 and is translated in different languages. The ICF model describes human functioning in terms of Body structure, Body function, Activities and Participation. The basic premise of the ICF is that it is *Universal*, that is applicable to all people irrespective of health condition or cultural context. ICF could be used for self‐ and/or proxy‐assessments (careers, professionals, family members).^124^, ^125^ Among items of oral functions, the following terms are defined as: Biting function (b5101): Functions of cutting into piercing or tearing off food with the front teeth; Chewing function (b5102): Functions of crushing, grinding and masticating food with the back teeth (eg molars); and Manipulation of food in the mouth (b5103): Functions of moving food around the mouth with the teeth and tongue. The extent of impairment is categorized with the following qualifiers as: 0 No impairment (category 0): means the person has no problem; Mild impairment (category 1): means a problem that is present less than 25% of the time, with an intensity which is tolerable and which happens rarely over the last 30 days; Moderate impairment (category 2): means that a problem is present less than 50% of the time, with an intensity which interferes in day to day life and which happens occasionally over the last 30 days; Severe impairment (category 3): means that a problem is present more than 50% of the time, with an intensity which partially disrupts day to day life and which happens frequently over the last 30 days; Complete impairment (category 4): means that a problem is present over 95% of the time, with an intensity that totally disrupts day to day life and happens every day over the last 30 days; Not specified (category 8): means there is insufficient information to specify the severity of the impairment; Not applicable (category 9): means it is inappropriate (eg chewing function for gastrectomised or tube‐fed patients).

##### Self‐ or proxy‐assessed masticatory function: Advantages

The subjective evaluation of masticatory function includes other aspects of mastication such as adaptational and psychological factors that cannot be measured with objective tests. These patient‐based outcomes are considered to be a useful screening method for assessing the masticatory function clinically with considerable cost and time savings.^126^, ^127^ They were mainly created for epidemiological purposes to detect changes in chewing and to provide actions aimed at caring for patients.^127^

##### Self‐ or proxy‐assessed masticatory function: Limitations

There is a poor correlation between objective evaluation of masticatory function and patient's perception.^17^, ^128^ Moreover, choosing an appropriate questionnaire instrument for a particular study is mandatory. Categorical answers, for instance, may not detect subtle differences, among subjects which may be clinically important.^127^ Measurements based on VAS, on the other hand, allows parametric statistical approaches when random variation is low. Furthermore, questionnaires that include specific food types are very much limited to a specific cultural, or socio‐economic context. For example, questions related to the consumption of meat or raw fish might be irrelevant in countries like India with a high prevalence of individuals with a vegetarian diet. Hence it seems advantageous to enquire about food textures.

##### Self‐assessed masticatory function: Clinical relevance

Epidemiological instruments as ICF are important to allow specific public health interventions. On the other hand, patient‐centred instruments are the best predictors of patient's choice of treatment, since they are also capable to detect significant differences between treatments.^127^ These questionnaires and interviews also help clinicians to better understand the effect of different treatments on patient's well‐being.

## 7 DISCUSSION

The increased interest in functional oral rehabilitation and in understanding the masticatory process, along with the confusing use of different terms to describe this process, created a demand for a new consensus on mastication terminology. We were aware of the difficulties in meeting such a challenge, but we tried to draw up consensus definitions to guide future studies. Our main goal was to reduce misuse of terms to facilitate comparisons among different studies evaluating masticatory function all over the world. We are also aware that mastication is an integral part in food processing, which is a prerequisite for safe and adequate swallowing. However, in this consensus paper, we intentionally focus on biting and chewing, and do not evaluate the terminology related to the swallowing process.

The outcome of mastication can be objectively evaluated using two different approaches with different research goals: the food bolus collected after a pre‐determined number of chewing cycles or the food bolus collected just before deglutition (at the ‘swallowing threshold’). In the first approach, the masticatory performance can be determined. The second approach provides information on the number of chewing cycles needed to prepare the food for swallowing and on the particle sizes of the chewed food bolus.

It was evident that the most confusing terms were ‘masticatory performance’ and ‘masticatory efficiency’, which were used interchangeably in many cases. Masticatory efficiency is calculated from the results of masticatory performance as determined after multiple numbers (at least two) of fixed chewing cycles.^16^, ^33^, ^34^, ^54^ Thus, masticatory efficiency is a more elaborate version of masticatory performance: it is based on several determinations of masticatory performance (Figure 1). Moreover, we pointed out that the term ‘masticatory performance’ applies for all the tests with a fixed number of chewing cycles. Thus, the term masticatory performance must be accompanied by a description of the method employed (Figure 1).

Another point of discussion was about the terms ‘chewing’ and ‘mastication’. The terms chewing performance and masticatory performance have been used interchangeably in the numerous studies of the masticatory process during the last decades, although most studies used the term masticatory performance.^16^, ^19^ The same holds for chewing efficiency and masticatory efficiency.^16^, ^33^, ^34^ According to the Glossary of Prosthodontic Terms, ‘mastication’ is defined as ‘the process of chewing food for swallowing and digestion’, whereas ‘chewing’ is ‘the deformation of food or other materials (for instance chewing gum, wax or Optosil) between the teeth and soft oral tissues’. In this sense, chewing would be part of mastication.^20^, ^32^ However, in a practical point of view, most studies consider chewing and mastication terms interchangeable, what is also confirmed in main dictionaries, for example Merriam‐Webster (US English) and Oxford (UK English).

The group discussed the ability of a specific test to objectively determine whether a subject or a group of subjects has or has not the capacity to form an acceptable food bolus, that is safe to swallow. It would be interesting to determine a standard cut‐off point as a ‘gold standard’ assessment of masticatory function. However, a variety of uncontrolled confounding factors may interfere with the masticatory assessment, jeopardising comparisons between different studies. One of these uncontrolled confounding factors relates to the characteristics of the test material employed to evaluate mastication. Food properties are relevant in determining masticatory performance and might be used to classify the employed methods, for example (a) solid relatively brittle food (nuts or artificial test foods related to Optosil), (b) solid less hard non‐brittle food: gummy jelly, (c) granules included in a capsule and (d) soft semi‐solid food: chewing gum or wax. Natural foods, for example carrot or peanuts, may differ in hardness among countries or regions, apart from seasonal variation.^129^ Moreover, differences in artificial test materials as product batch, methods of storage, product versions or even in the manipulation of the material may alter the rheological properties of the final product.^113^, ^130^, ^131^, ^132^

Other confounding factors comprise differences in the material processing. It might include differences between the optical or sieving analysis, the use of professional vibrating machines or regular plaster vibrators or even differences in the shaking time or the instructions of the vibrating machine. At last, a particular uncontrolled factor is related to the sieving method, especially when subjects with poor masticatory performance are evaluated. In such cases, the particles remain close to the initial size and small differences are difficult to be detected.^59^, ^133^ An optimal initial particle edge size should be determined in advance, based on the sieve aperture and the way of data processing of cumulative weight data from sieving.^54^

The mastication process may belong to one of the three following conditions: totally healthy, moderately impaired and totally impaired, corresponding to masticatory capacity, compensatory adaptation and masticatory incapacity.^134^, ^135^ Capacity means that mastication is perfectly achieved. Compensatory adaptation occurs when mastication is slightly disturbed, but the individual concerned can implement a physiological adaptation, mainly an increase in the number of cycles, that makes a normal bolus. Incapacity/deficiency means the function is largely deficient, because the individual fails to make a proper food bolus. In such subjects, adaptability is overstretched; they are unable to masticate correctly as shown by the final food bolus. In the latter group, the main subjects’ strategies to feed themselves despite their incapacity are changing diets and swallowing unchewed food. Negative nutritional consequences and/or excessive workload inflicted on the digestive tract are likely. Compensatory adaptation or incapacity can be found in many settings such as craniofacial dysmorphia, neurological diseases, traumatic or surgical sequelae, temporomandibular disorders and other conditions leading to occlusal changes including partial or complete edentulousness.

Although poorly considered and epidemiologically insufficiently documented, mastication incapacity can be expected in large groups of persons. For example, in persons with neuromotor and cognitive disorders such as Parkinson disease, stroke, congenital or acquired brain damage and other neurological disorders. Also, some groups of elderly showed mastication deficiencies values between 20% to 50% with a strong influence of the number of missing teeth.^135^ A value of 30.5% total or partial incapacity was also reported with participants aged 20‐59 years in a cross‐sectional study.^120^

The group expressed the need to concentrate research on finding one or several tests to determine objectively if a subject or a group of subjects has or has not the capacity to make an acceptable food bolus. It would be interesting to determine a standard cut‐off point, a marker, for masticatory function like glycaemia, creatinine clearance, blood cell counts, globulin or lipids blood concentrations offer cut‐off values/markers for hepatic, renal, blood, immunologic functions or lipid metabolism. The proposal of a masticatory normative indicator (MNI) for carrots^47^ and Optosil^136^ was a first step in this direction. The permanence of the same mastication frequency for a given individual, masticating a given food differing only by hardness is another example. Because relatively elaborate laboratory procedures are mandatory for these markers, their use will be restricted to research. More casual markers offering a cut‐off value are expected in the future. Of course, and like many other tests which have been elaborated in other medical fields, before reaching a consensus, a variety of uncontrolled confounding factors will interfere with the test finalisation. Comparisons between different proposed tests will be difficult. Possible confounding factors relate to the characteristics of the test material, the choice and the standardisation of the methods. Validations will have to be carried out. To reach a consensus, the desired test(s) must be based on a reproducible method and most of all, easy to use for clinicians either in private clinical practice or in dental facilities. Such a test offering a cut‐off value will be of great clinical significance to assess capacity versus incapacity/deficiency.

Besides objectively measuring one's masticatory performance, the evaluation of the subjective components of mastication is also subjected to confusing terminology. The term ‘masticatory ability’ can be considered as a general definition of the chewing process.^17^, ^83^ However, it has more frequently been associated to self‐assessed masticatory function studied by interviewing subjects on their oral function.^19^ In order to avoid misuse or confusion, we adopted the term ‘Self‐assessment of masticatory function’. Self‐reported perceptions have the limitations to be over‐estimated, especially in elderlies and complete denture wearers.^19^ Objective and subjective methods show weak correlations.^137^ However, information regarding subject's food avoidance, personal preferences, nutritional habits, among others can only be obtained by questionnaires or interviews. Thus, objective and subjective analyses are complementary and should be applied together.

Recently, an interesting approach was proposed by Jeltema, Beckley and Vahalik^138^, ^139^ for consumer segmentation. By observing and surveying more than 500 people, they found that individuals have a preferential way to process food in their mouths (Mouth Behavior) and these preferences drive texture preference and food choice. They suggested that consumers fall in roughly four major Mouth Behavior groups (‘Chewers, ‘Crunchers’, ‘Smooshers’ and ‘Suckers’) and can be easily categorised. They observed that four groups fell into two major modes of mouth actions. Chewers and Crunchers used their teeth to breakdown foods, whereas Smooshers and Suckers preferred to manipulate it between the tongue and the roof of the mouth.^138^, ^139^

The current consensus intended to serve as a guideline for future researches. As such, there is no guarantee about its implementation. Periodic reviews of definitions are encouraged and updates made when appropriate. We hope this consensus statement can be widely supported and adopted in order to standardise masticatory terminology and guide advances in future studies.

## 8 CONCLUSION

In this consensus review, the definitions of the most commonly used terms and techniques related to masticatory function assessment were discussed and updated. Advantages, limitations and relevance of each method are also revised, based on current concepts published and on experts’ opinions. The suggested terminology will facilitate less ambiguous communication between researchers, clinicians and their patients. It will also enable better documentation and interpretation of research findings and clinical observations. A regular update of the terms is advisable in order to reflect up‐to‐date scientific standard.

## CONFLICT OF INTEREST

The authors declare no conflict of interest.

## AUTHOR CONTRIBUTION

Gonçalves TMSV is General coordinator of the consensus. She was responsible for monitoring all stages, managing the manuscript from the conception and design to data review, analysis and interpretation. She substantially contributed to the scientific writing of the article and general supervision of the manuscript intellectual review and paper submission. Schimmel M – He was a sub‐coordinator of the consensus with substantial contribution to the conception and study design, actively participating during data acquisition and interpretation. He was also involved in the manuscript submission. Van der Bilt A – He had contributed for data synthesis and analysis, scientific writing of the article and intellectual reviewing of the manuscript. Chen J – He had participated in data analysis and drafting of the manuscript, revising it critically. Van der Glas H – He had participated in data analysis and drafting of the manuscript, revising it critically. Kohyama K – She had participated in data analysis and drafting of the manuscript, revising it critically. Hennequin M – She had participated in data analysis and drafting of the manuscript, revising it critically. Peyron MA – She had participated in data analysis and drafting of the manuscript, revising it critically. Woda A – He had participated in data analysis and drafting of the manuscript, revising it critically. Leles CR – He had participated in data analysis and drafting of the manuscript, revising it critically. Pereira LJ – He was also a sub‐coordinator of the consensus. He substantially contributed to the drafting of the manuscript and data analysis and interpretation. He also worked on the critical review of the paper and final approval of the version submitted.

### PEER REVIEW

The peer review history for this article is available at https://publons.com/publon/10.1111/joor.13161.

## Data Availability

Data sharing is not applicable to this article as no new data were created or analysed in this study.
